# Follow-up score, change score or percentage change score for determining clinical important outcome following surgery? An observational study from the Norwegian registry for Spine surgery evaluating patient reported outcome measures in lumbar spinal stenosis and lumbar degenerative spondylolisthesis

**DOI:** 10.1186/s12891-018-2386-y

**Published:** 2019-01-18

**Authors:** Ivar Magne Austevoll, Rolf Gjestad, Margreth Grotle, Tore Solberg, Jens Ivar Brox, Erland Hermansen, Frode Rekeland, Kari Indrekvam, Kjersti Storheim, Christian Hellum

**Affiliations:** 1 Kysthospitalet in Hagevik, Orthopedic Clinic, Haukeland, University Hospital, Hagaviksbakken 25, 5217 Hagevik, Bergen, Norway; 2 Department of Clinical Medicine, University of Bergen, Christies gate 6, 5007 Bergen, Bergen, Norway; 3 The Norwegian Registry for Spine Surgery (NORspine), Northern Norway Regional Health Authority, Postboks 20, 9038 Tromsø, Bodø, Norway; 4 Research Department, Division of Psychiatry, Haukeland University Hospital, Sanviksleitet 1, 5036 Bergen, Bergen, Norway; 5 Department of Physical Medicine and Rehabilitation, Oslo University Hospital, PB 4950 Nydalen, 0424 Oslo, Oslo Norway; 6 Department of Neurosurgery, University Hospital of Northern Norway, Sykehusvegen 38, 90919 Tromsø, Tromsø, Norway; 7 Research and Communication Unit for Musculoskeletal Health (FORMI), Oslo University Hospital, PB 4950 Nydalen, 0424 Oslo, Oslo Norway; 8 Department of Orthopedic Surgery, Ålesund Hospital, Møre and Romsdal Hospital Trust, Ålesund, Norway; 9 Division of Orthopaedic Surgery, Oslo University Hospital, 4950 Nydalen, 0424 Oslo, PB Norway; 10 Faculty of Health Science, OsloMet – Oslo Metropolitan University, PO box 4 St. Olavs plass, 0130 Oslo, Oslo Norway

**Keywords:** Lumbar spinal stenosis (LSS), Lumbar degenerative spondylolisthesis (LDS), Patient reported outcome measures (PROMs), Oswestry disability index (ODI), Leg pain, Back pain, Success criteria, Minimal clinically important difference (MCID)

## Abstract

**Background:**

Assessment of outcomes for spinal surgeries is challenging, and an ideal measurement that reflects all aspects of importance for the patients does not exist. Oswestry Disability Index (ODI), EuroQol (EQ-5D) and Numeric Rating Scales (NRS) for leg pain and for back pain are commonly used patients reported outcome measurements (PROMs). Reporting the proportion of individuals with an outcome of clinical importance is recommended. Knowledge of the ability of PROMs to identify clearly improved patients is essential. The purpose of this study was to search cut-off criteria for PROMs that best reflect an improvement considered by the patients to be of clinical importance.

**Methods:**

The Global Perceived Effect scale was utilized to evaluate a clinically important outcome 12 months after surgery. The cut-offs for the PROMs that most accurately distinguish those who reported ‘completely recovered’ or ‘much improved’ from those who reported ‘slightly improved’, unchanged’, ‘slightly worse’, ‘much worse’, or ‘worse than ever’ were estimated. For each PROM, we evaluated three candidate response parameters: the (raw) follow-up score, the (numerical) change score, and the percentage change score.

**Results:**

We analysed 3859 patients with Lumbar Spinal Stenosis [(LSS); mean age 66; female gender 50%] and 617 patients with Lumbar Degenerative Spondylolisthesis [(LDS); mean age 67; 72% female gender]. The accuracy of identifying ‘completely recovered’ and ‘much better’ patients was generally high, but lower for EQ-5D than for the other PROMs. For all PROMs the accuracy was lower for the change score than for the follow-up score and the percentage change score, especially among patients with low and high PROM scores at baseline.

The optimal threshold for a clinically important outcome was ≤24 for ODI, ≥0.69 for EQ-5D, ≤3 for NRS leg pain, and ≤ 4 for NRS back pain, and, for the percentage change score, ≥30% for ODI, ≥40% for NRS leg pain, and ≥ 33% for NRS back pain. The estimated cut-offs were similar for LSS and for LDS.

**Conclusion:**

For estimating a ‘success’ rate assessed by a PROM, we recommend using the follow-up score or the percentage change score. These scores reflected a clinically important outcome better than the change score.

## Background

The success of surgical treatment of spinal degenerative disorders is basically determined by reduction of pain and improvement of function. In clinical studies, treatment effects are most commonly assessed by patient reported outcome measures (PROMs) [1–5]. Widely used PROMs for evaluating outcomes after surgery for lumbar spinal stenosis (LSS) with and without degenerative spondylolisthesis (LDS) are the Oswestry Disability Index (ODI) [1, 2, 4, 5], the numeric rating scales (NRS) for leg- and back pain [1, 6–9], and a generic measure of health-related quality of life such as the EQ-5D [8–10]. However, these outcome measures do not necessarily cover all areas of interest to the patient. Even though items like personal care and walking distance are addressed by the ODI, more specific disabilities such as problems with personal hygiene, posture imbalance and slow walking speed may not be detected.

Due to the frequent use of PROMS, the statistical application and the interpretation of the clinical importance of the outcomes should be evaluated [11]. The clinical effect of a treatment is usually only presented as the mean change from baseline to follow-up [1, 4, 5]. However, a statistically significant mean group difference does not necessarily provide meaningful clinical information when comparing two methods. A large improvement in a few individuals in one of the treatment groups can dramatically enhance the mean change of the group, even if the majority had no improvement or even a slight worsening of their complaints [11, 12]. Rather than discussing the relevance of mean changes alone, the proportion of individuals with a clinically relevant reduction in pain and disability (i.e., a ‘success’ rate) can be employed as a comprehendible metric for patients and physicians to use in clinical decision-making [11–13].

To calculate ‘success’ rates assessed by PROMs, we need criteria that reflect the patients’ perceptions of important benefits following operations [11–13]. The Minimal clinical important difference (MCID) was the first metric developed for this purpose [14, 15]. Minimal important changes (MIC) [16], a substantial clinical benefit [17] and a satisfactory symptom state [18, 19] are other metrics developed to distinguish whether patients have achieved a clinically important effect of treatment or not. Several authors have pointed out the great variability and diversity of such thresholds [12, 20, 21], which may be caused by the heterogeneity in the populations studied [22]. The objective of the present study was to estimate the thresholds for ODI, EQ-5D and NRS leg- and back pain that best identify the patients who perceived a clinically important outcome following surgery for LSS and LDS. Receiver Operating Characteristic (ROC) analyses were evaluated to explore how accurately ‘success’ assessed by a single question on the Global Perceived Effect (GPE) scale [23] would be reflected in the PROMs. Despite limited evidence for the validity of the GPE scale [12, 24], it is widely used [17, 18, 25–28] and recommended [12, 29] in such analyses. For each PROM three alternative response parameters were evaluated: the follow-up score, the change score and the percentage change score. LSS and LDS were analysed separately.

## Methods

### Study population

Data were obtained from the Norwegian Registry for Spine Surgery (NORSpine). NORSpine is a government-funded, comprehensive, clinical registry for quality control and research. The registry receives no funding from the industry. Informed consent is obtained from all patients. The patient form consists of PROMs completed before surgery (baseline) and at 3- and 12-month follow-up. During the hospital stay, data concerning diagnosis, treatment and comorbidity were recorded by the surgeons on a standard form.

Inclusion criteria: (1) Patients registered in NORSpine in the period 2007–2013; (2) Patients assessed by the surgeon to have spinal stenosis with or without degenerative spondylolisthesis; (3) Patients operated with a decompression procedure or with decompression in combination with posterior fusion. Patients with a former operation at index level were excluded.

### Patient reported outcome measures (PROMs)

1. The Oswestry Disability Index (ODI) V.2.0 [30, 31] has been translated and validated for application among Norwegian patients [32]. It is found to be an appropriate instrument for assessing treatment outcome in patients with spinal stenosis with and without a degenerative spondylolisthesis [33]. It is a self-reported instrument comprising 10 questions about pain related disability in activities of daily life. The sum score ranges from 0 (no disability) to 100 points (bedridden).
2. The EuroQol (EQ-5D) [34] is a generic measurement for assessing health-related quality of life. It evaluates mobility, self-care, usual activity, pain/discomfort and anxiety/discomfort. For each component the patients can choose between three answers; none, mild to moderate, and severe. This gives 3^5^ = 243 possible sets of answers, and each unique combination corresponds to a value between − 0.59 and 1.0, where 1.0 represents perfect health.
3. Numeric Rating Scale (NRS) for back- and leg pain assesses self-reported pain level in the last week ranging from 0 (no pain) to 10 (worst conceivable pain) [30].
4. Global Perceived Effect (GPE) is a single question measuring patient-rated assessment of treatment outcome [23]. The patient may choose between seven response alternatives: ‘completely recovered’, ‘much improved’, ‘slightly improved’, unchanged’, ‘slightly worse’, ‘much worse’, and ‘worse than ever’.

### Definition of ‘success’ according to GPE scale

Patients who rated themselves as ‘completely recovered’ or ‘much improved’ on the GPE scale (the anchor) at 12-month follow-up were considered to have gained a clinically important outcome following the surgery (‘success’), whereas patients that replied ‘slightly improved’, unchanged’, ‘slightly worse’, ‘much worse’, and ‘worse than ever’ were considered to have not benefited from their operation (‘non-success’) [12, 17, 18, 35].

### Statistics

For each PROM three alternative response parameters were evaluated: 1) the (raw) follow-up score; 2) the (numerical) change score (i.e., the absolute change from baseline to follow-up); 3) the percentage change score (i.e., the change score as a percentage of the baseline score). In order to evaluate whether ‘success’ on the GPE scale (the anchor) would be reflected in a PROM, Receiver Operating Characteristics (ROC) [36] curve analyses were performed. Analogue to a diagnostic test, the sensitivity refers to the probability of detecting a condition. In the present setting it refers to the probability of correctly classifying an individual replying ‘completely recovered’ or ‘much improved’ (GPE) as a ‘success’ when assessed by a PROM. Correspondingly, the specificity refers to the probability of correctly classifying a patient reporting less than ‘completely recovered’ or ‘much improved’ as a ‘non-success’. Depending on the level of a cut-off, the sensitivity and specificity will vary. A ROC curve was made by plotting the sensitivity against 1 minus the specificity, for all possible cut-off values for ‘success’. The cut-off that maximized the proportion of correctly classified patients according to the anchor was chosen as the threshold for ‘success’. If more than one cut-off value maximized the percentage of correct classification we prioritized the relation between sensitivity and specificity that balanced the ratio between false negatives and false positives [13, 36]. If possible, still with the assumption of maximum correct classification and a balanced false negatives/false positives ratio, we intended to choose common cut-off values for LSS and LDS.

For all PROMs, the area under the ROC curves (AUC) with 95% confidence interval (CI) was estimated for the alternative response parameters. The AUC describes the test’s accuracy in correctly classifying a case according to the anchor – the larger the AUC, the greater the accuracy of the test. The AUC is classified as ‘excellent’ from 1.0 to 0.90, ‘good’ from 0.90 to 0.80, ‘fair’ from 0.80 to 0.70, ‘poor’ from 0.70 to 0.60, and ‘failed’ from 0.60 to 0.50 [37].

Since cut-off values for clinical improvement tend to be dependent on the baseline level of a measurement [26], a sensitivity analysis was performed. For each of the estimated cut-off values the percentage of correct classification was calculated for patient groups with low, medium, and high baseline scores respectively. The split values were chosen to ensure equal proportions of patients in each group (tertiary split). For ODI the split values between groups were 32 and 46 points, for EQ-5D they were 0.1 and 0.6. For NRS leg- and back pain the low baseline group had scores of 1–5, the medium baseline group, 6–7 and the high baseline group, 8–10.

Baseline characteristics and PROMs were reported as means and standard deviations of continuous variables and as percentages of categorical variables. The mean 12-month follow-up scores and the mean changes from baseline to follow-up were assessed against the categories of the GPE scale. To evaluate the predictive validity of PROMs, correlations between the response on the GPE scale and the PROMs were analysed using the Spearman rank coefficient.

In a previous study from NORSpine, no differences in outcome were found when comparing compliers and non-compliers at follow-up [38]. We therefore assumed that missing data were comparable to data from those who answered, and performed the analysis based on the listwise deletion method [39].

The statistical analyses were performed using the Statistical Package for Social Sciences (SPSS) version 23.0 and by Stata version 14.0.

## Results

**Fig. 1:**
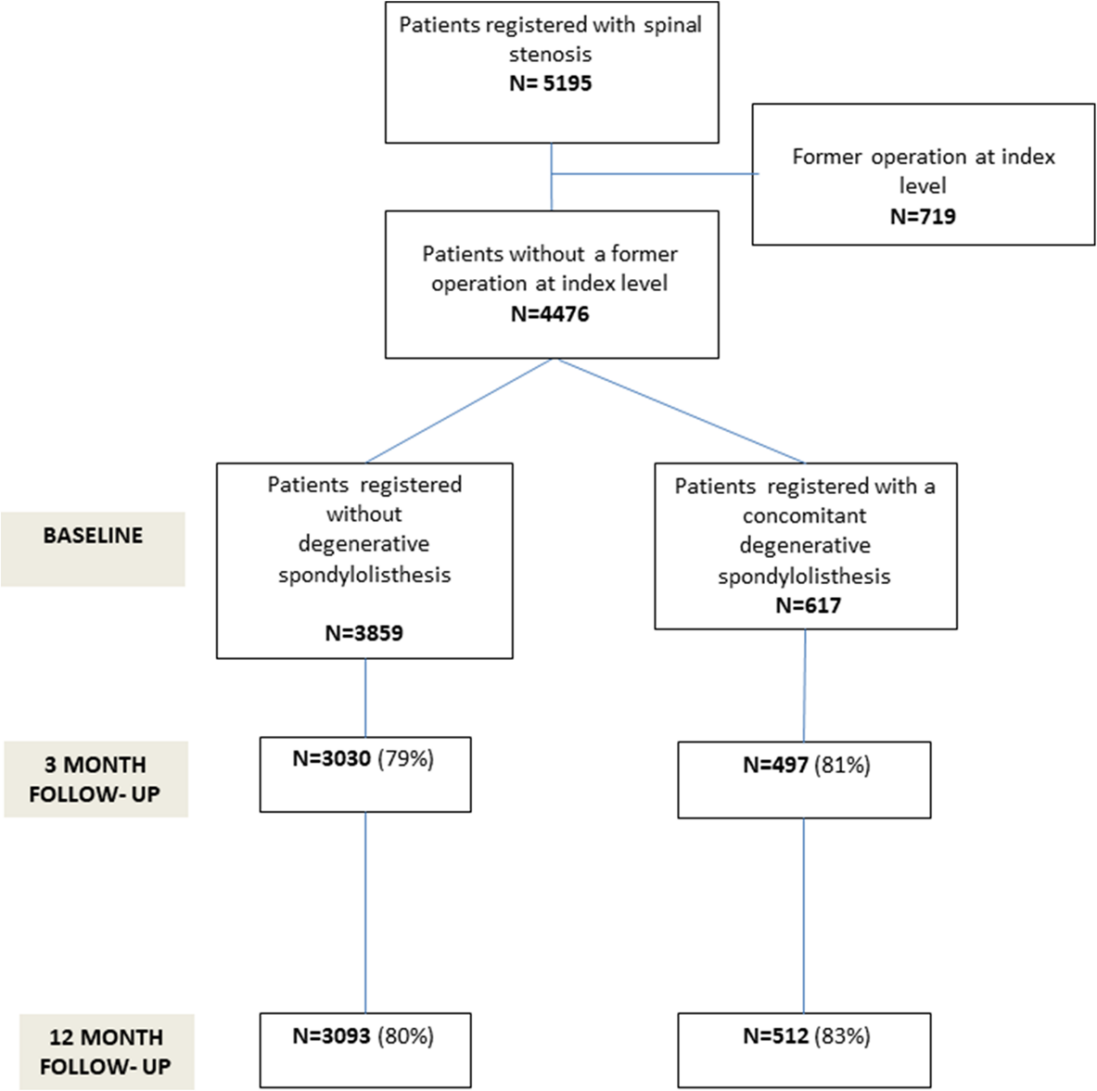
Flow chart for patients registered with spinal stenosis in NORSpine in the period 2007–2013

Of 5238 eligible patients from 32 clinics, 4476 met the inclusion criteria. Of these, 617 had a degenerative spondylolisthesis. At 12-month follow-up, 3093 with LSS and 517 with LDS had answered the questionnaire, a follow-up rate of 81% (Fig. 1).

The mean age (±SD) was 66 (±11) years for LSS and 67 (±10) years for LDS, and the percentage of females was 50 and 72%, respectively. Further patient demographics and surgical data are presented in Table 1.

**Table 1:** Patient demographics and surgical data for patients operated for spinal stenosis and for degenerative spondylolisthesis

	Spinal stenosis		Degenerative spondylolisthesis	
	N		N	
Age; Yr ± SD	3858	66 ± 11	617	67 ± 10
Female, no (%)	3859	1919 (50%)	617	444 (72%)
ASA level (1–4); Mean ± SD	3759	2.0 ± 0.6	608	2.0 ± 0.5
ASA level 1, no (%)		681 (18%)	82 (13%)	
ASA level 2, no (%)		2349 (61%)	429 (71%)	
ASA level 3, no (%)		753 (19%)	97 (16%)	
ASA level 4, no (%)		12(0.3%)	0	
Body Mass Index; Mean (SD)	3547	27 ± 4	560	27.0 ± 5
Smokers, no (%)	3808	877 (23%)	609	115 (19%)
Laminectomy, no (%)	3859	1024 (27%)	617	239 (39%)
Midline preserving decompression, no (%)	3859	2835 (73%)	617	378 (61%)
Fusion, no (%)	3859	214 (6%)	617	297 (48%)
ODI; Mean (SD)	3837	40 ± 15	617	41 ± 15
EQ-5D; Mean (SD)	3535	0.37 ± 0.32	564	0.34 ± 0.32
NRS leg pain; Mean (SD)	3559	6.6 ± 2.2	569	6.7 ± 2.2
NRS back pain; Mean (SD)	3597	6.4 ± 2.2	573	6.9 ± 2.1

*N* number of patient with data for the evaluated parameter

The mean (±SD) ODI changed from 40 (±15) at baseline (Table 1) to 23 (±18) at 12-month follow-up (Table 2) for LSS, and from 41 (±15) to 22 (±18) for LDS. Respectively for LSS and LDS, EQ-5D changed from 0.37 (±0.32) to 0.64 and from 0.34 (±32) to 0.67, NRS leg pain from 6.6 (±2.2) to 3.5 (±3.0) and 6.7 (±2.2) to 3.2 (±2.9) and NRS back pain from 6.4 (± 2.2) to 3.8 (±2.8) and 6.9 (±2.2) to 3.6 (±2.8). On the GPE-scale 58 and 65% replied that they were ‘completely recovered’ or ‘much improved’ (LSS and LDS, respectively). The Spearman rank coefficients between the GPE ratings and the 12-month follow-up measures were 0.77 and 0.78 for ODI, 0.73 and 0.78 for EQ-5D, 0.72 and 0.68 for NRS leg pain and 0.76 and 0.78 for NRS back pain, respectively for LSS and LDS; *p* < 0.001 for all correlations (Table 2).

**Table 2:** Follow-up scores and the change scores for PROMs according to the GPE-scale

		Spinal stenosis								Degenerative spondylolisthesis							
		N	(%)	1 year Follow-up Mean (SD)		Spear man’s rho	Change score Mean (SD)		Spearman’s rho	N	(%)	1 year Follow- up Mean (SD)		Spearman’s rho	Change score Mean (SD)		Spear- man’s rho
	All	3060		23	(18)	0.77 *	16	(18)	0.66*	509		22	(18)	0.78*	19	(17)	0.64*
O	Compl.recovered	599	(20%)	4	(9)		32	(16)		117	(23%)	4	(7)		33	(15)	
D	Much improved	1176	(38%)	17	(12)		21	(15)		213	(42%)	17	(13)		23	(14)	
I	Slightly improved	658	(21%)	32	(12)		9	(13)		105	(21%)	36	(13)		9	(12)	
	Unchanged	283	(9%)	38	(13)		0	(10)		33	(6%)	38	(14)		5	(13)	
	Slightly worse	181	(6%)	42	(13)		0	(12)		21	(4%)	41	(13)		3	(13)	
	Much worse	117	(4%)	49	(12)		−3	(12)		11	(2%)	51	(11)		−8	(13)	
	Worse than ever	46	(2%)	59	(15)		−11	(12)		9	(2%)	57	(17)		−7	(15)	
	Missing	799								108							
	All	2464		0.64	(0.31)	0.73*	0.25	0.36	0.50*	419		0.67	(0.30)	0.78*	0.32	(0.34)	0.48*
E	Compl.recovered	463	(19%)	0.92	(0.15)		0.47	(0.32)		97	(23%)	0.93	(0.16)		0.51	(0.30)	
Q	Much improved	945	(38%)	0.74	(0.17)		0.34	(0.32)		175	(42%)	0.75	(0.16)		0.37	(0.32)	
-	Slightly improved	543	(22%)	0.55	(0.26)		0.19	(0.33)		89	(21%)	0.46	(0.29)		0.18	(0.31)	
5	Unchanged	230	(9%)	0.41	(0.31)		0.03	(0.29)		26	(6%)	0.40	(0.30)		0.08	(0.33)	
D	Slightly worse	148	(6%)	0.33	(0.32)		0.00	(0.32)		17	(4%)	0.36	(0.30)		0.13	(0.29)	
	Much worse	100	(4%)	0.15	(0.23)		0.15	(0.32)		8	(2%)	0.30	(0.34)		0.02	(0.08)	
	Worse than ever	35	(1%)	0.04	(0.22)		0.24	(0.37)		7	(2%)	0.08	(0.24)		0.03	(0.11)	
	Missing	1395								198							
L	All	2988		3.5	(3.0)	0.72*	3.1	(3.3)	0.63*	493		3.2	(2.9)	0.68*	3.5	(3.2)	0.58*
E	Compl.Recovered	580	19%	0.6	(1.5)		5.9	(2.5)		112	(23%)	0.6	(2.2)		6.0	(2.5)	
G	Much improved	1159	39%	2.5	(2.2)		4.0	(2.7)		208	(42%)	2.6	(2.2)		4.0	(2.7)	
	Slightly improved	640	21%	4.9	(2.2)		1.8	(2.6)		102	(20%)	4.8	(2.4)		1.8	(2.6)	
P	Unchanged	275	9%	6.3	(2.1)		0.1	(2.3)		33	(7%)	6.1	(4.7)		0.4	(2.2)	
A	Slightly worse	176	6%	6.4	(2.1)		0.7	(2.6)		18	(4%)	5.2	(3.0)		1.0	(2.9)	
I	Much worse	114	4%	7.5	(2.1)		−0.5	(2.6)		11	(2%)	6.6	(2.3)		0.4	(3.8)	
N	Worse than ever	44	1%	7.7	(2.1)		−0.4	(2.9)		9	(2%)	7.8	(1.9)		0.0	(2.1)	
	Missing	871								124							
B	All	3033		3.8	(2.8)	0.76*	3.3	(2.9)	0.62*	507		3.6	(2.8)	0.78*	3.3	(2.9)	0.64*
A	Compl. recovered	592	20%	0.6	(1.4)		5.4	(2.5)		117	(23%)	0.7	(2.0)		5.8	(2.5)	
C	Much improved	1171	38%	3.0	(2.0)		3.2	(2.5)		214	(42%)	3.0	(2.0)		3.7	(2.5)	
K	Slightly improved	648	21%	5.2	(1.9)		1.4	(2.3)		105	(21%)	5.7	(1.7)		1.6	(1.8)	
	Unchanged	278	9%	6.5	(2.0)		0.5	(2.0)		32	(6%)	6.0	(2.0)		1.4	(2.1)	
P	Slightly worse	182	6%	6.7	(1.8)		0.1	(2.0)		20	(4%)	6.7	(1.6)		0.3	(1.6)	
A	Much worse	116	4%	7.4	(2.1)		−0.1	(2.2)		11	(2%)	7.3	(2.19		−0.2	(1.3)	
I	Worse than ever	46	2%	8.3	(1.9)		−0.8	(2.3)		8	(2%)	8.5	(1.3)		−0.4	(1.4)	
N	Missing	826								110							

Mean 1 year follow-up scores and mean change scores from baseline to follow-up for ODI, EQ-5D, NRS leg pain, and NRS back pain [positive values indicate decreased disability (ODI), improved health-related quality of life (EQ-5D), and reduced pain (NRS)]. Results are given for all patients, and for patients stratified according to the Global Perceived Effect (GPE) scale. The association between the outcome measurements and the GPE responses are given by Spearman’s rank correlation coefficients (Spearman’s rho)

*p<0.005

**Fig. 2:**
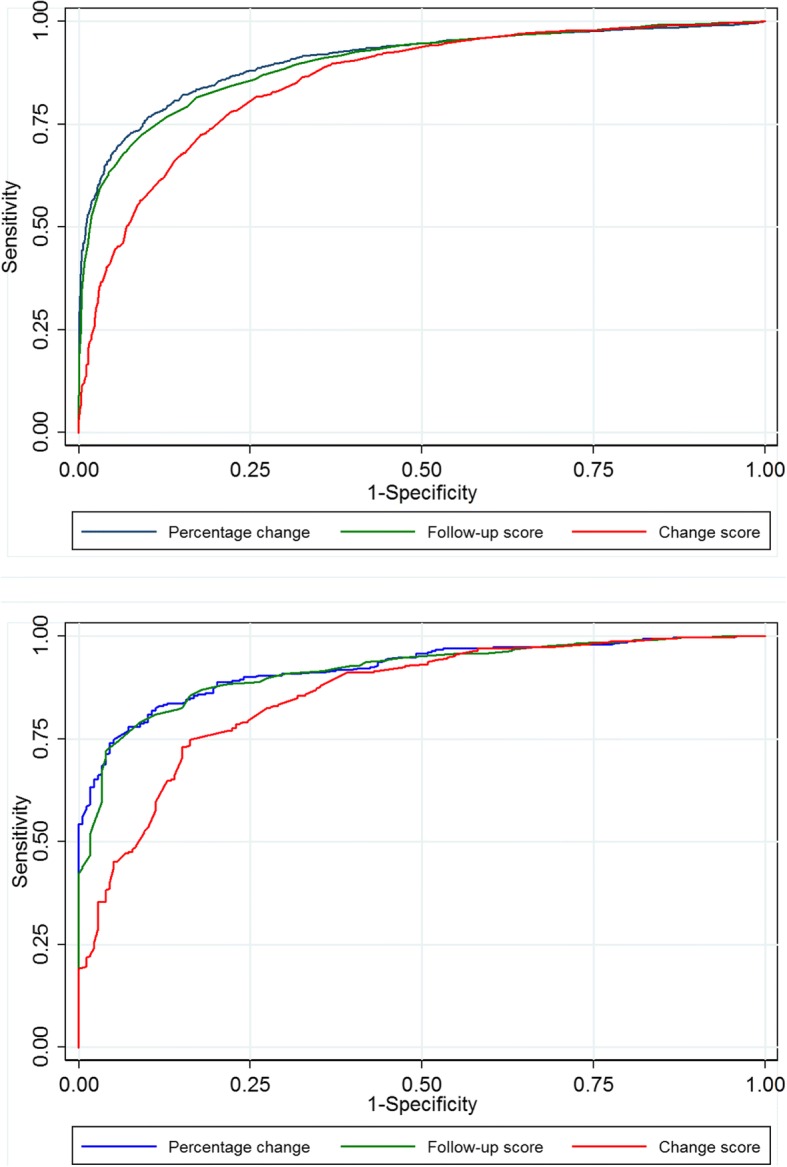
Receiver Operating Characteristic curves for ODI. Legend: The closer the curve is in the upper left corner, the higher accuracy for determining whether a patients is cured (‘completely recovered’ or ‘much improved’) or not. 2**a**. Spinal stenosis; 2**b**. Degenerative spondylolisthesis

**Fig. 3:**
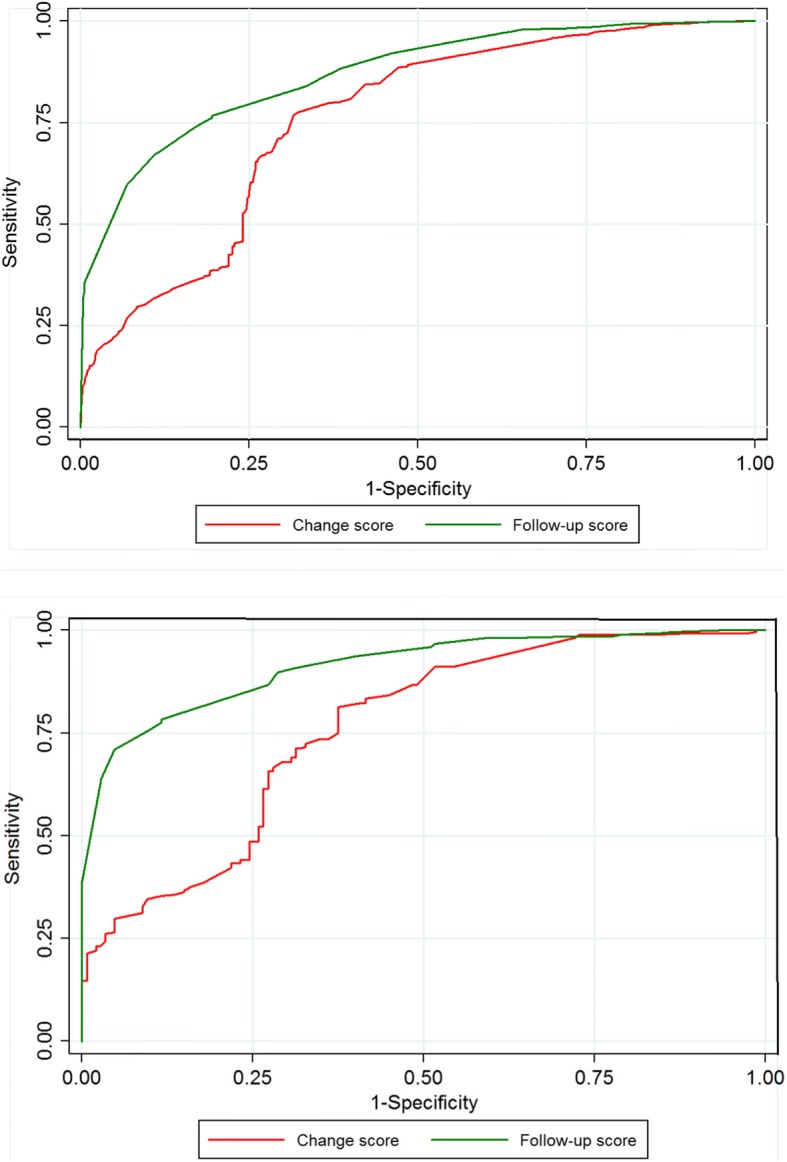
Receiver Operating Characteristic curves for EQ-5D. Legend: The closer the curve is in the upper left corner, the higher accuracy for determining whether a patients is cured (‘completely recovered’ or ‘much improved’) or not. 3**a**. Spinal stenosis; 3**b**. Degenerative spondylolisthesis

**Fig. 4:**
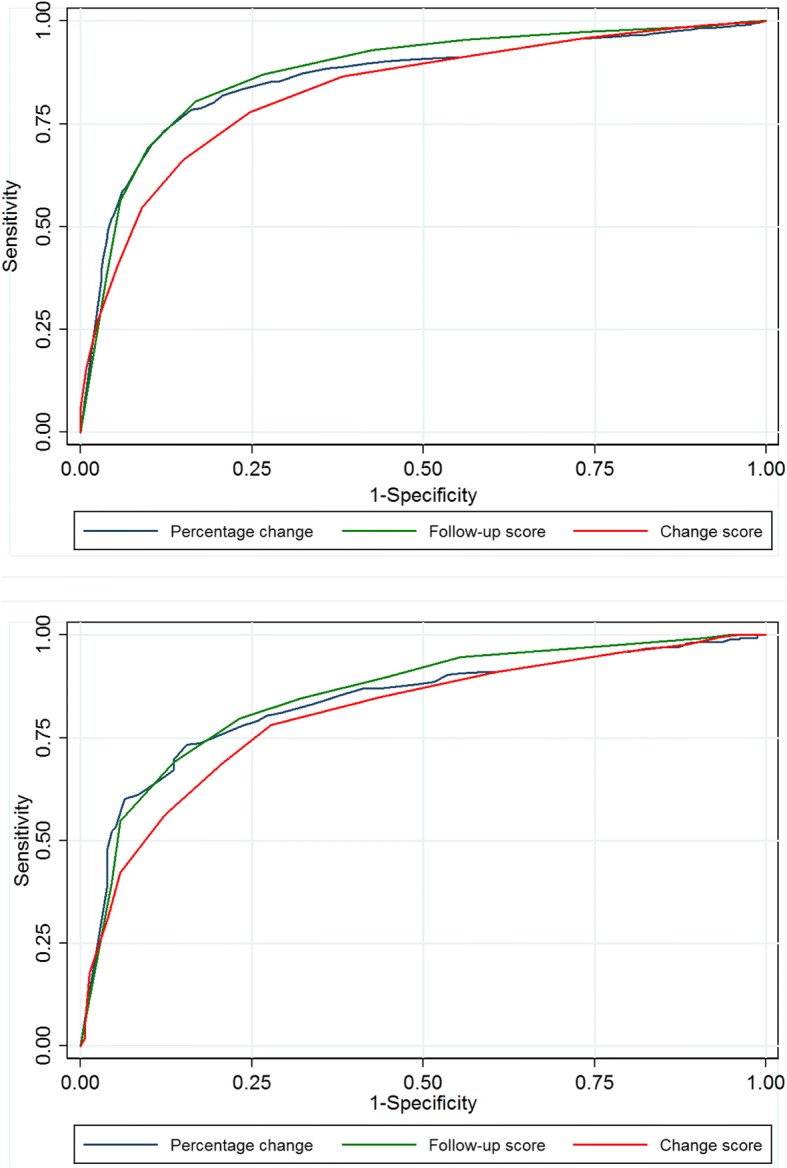
Receiver Operating Characteristic curves for NRS leg pain. Legend: The closer the curve is in the upper left corner, the higher accuracy for determining whether a patients is cured (‘completely recovered’ or ‘much improved’) or not. 4**a**. Spinal stenosis; 4**b**. Degenerative spondylolisthesis

**Fig. 5:**
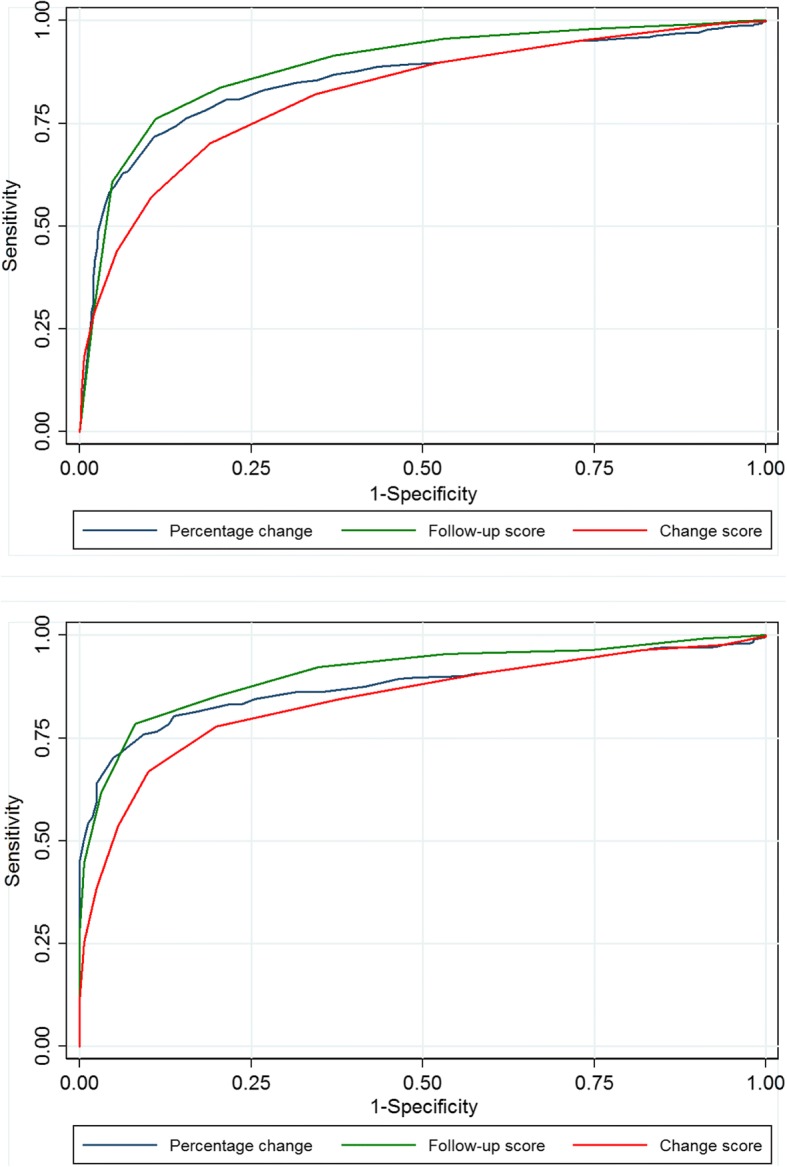
Receiver Operating Characteristic curves for NRS back pain. Legend: The closer the curve is in the upper left corner, the higher accuracy for determining whether a patients is cured (‘completely recovered’ or ‘much improved’) or not. 5**a**. Spinal stenosis; 5**b**. Degenerative spondylolisthesis

Figures 2, 3, 4 and 5 show the ROC curves for each of the response parameters for ODI, EQ-5D and NRS leg- and back pain. For all PROMs the graphs for the follow-up scores and the percentage change scores illustrate larger areas under the curves (AUC) than for the (numerical) change scores, indicating that the change scores were less accurate in matching ‘successes’ from the GPE scale.

In general, the computed AUC showed good or excellent test accuracy (AUC from 0.82 to 0.92) for the three alternative scores for all measurements except for the EQ-5D’s change score [AUC = 0.76 (fair accuracy)]. However, for all PROMs, the AUC was generally lower for the change scores than for the follow-up scores and the percentage change scores, and in most cases this difference was statistically significant (i.e., without overlap of the 95% CI (Table 3). For LSS, the AUC for ODI was 0.90 (95% CI 0.89–0.91) for the follow-up score, 0.86 (95% CI 0.84–-0.87) for the numerical change score and 0.91(95% CI 0.90–0.92) for the percentage change score, and, respectively, 0.92 (95% CI 0.89–0.94), 0.86 (95% CI 0.82–0.89) and 0.92 (95% CI 0.90–0.94) for LDS. The AUCs for all PROMs are listed in Table 3.

**Table 3:** ROC analyses for determining AUC (95% CI) and for estimating cut-off values for ‘success

		ODI				EQ-5D				NRS	Leg pain			NRS	Back pain	
	AUC	(95% CI)	Max corr. Class	Cut-off	AUC	(95% CI)	Max corr. Class	Cut-off	AUC		Max corr. Class.	Cut-off	AUC		Max corr. class.	Cut-off
Spinal stenosis																
Follow-up score (points)	0.90	(0.89–0.91)	82%	≤24	0.87	(0.85–0.88)	78%	0.692	0.87	(0.86–0.89)	81%	≤4	0.89	(0.87–0.90)	82%	≤4
Change score (points)	0.86	(0.84–0.87)	78%	≥13	0.76	(0.74–0.78)	73%	0.105	0.83	(0.82–0.85)	77%	≥3	0.82	(0.81–0.84)	75%	≥2
Percentage change (%)	0.91	(0.90–0.92)	83%	≥30					0.86	(0.85–0.88)	81%	≥40	0.86	(0.84–0.87)	79%	≥33
Degenerative Spondylolisthesis																
Follow-up score (points)	0.92	(0.89–0.94)	85%	≤24	0.92	(0.89–0.94)	84%	≥0.692	0.86	(0.82–0.89)	79%	≤3	0.90	(0.88–0.93)	83%	≤4
Change score (points)	0.86	(0.82–0.89)	80%	≥13	0.76	(0.76–0.81)	76%	≥0.105	0.81	(0.77–0.91)	76%	≥3	0.84	(0.81–0.88)	79%	≥3
Percentage change (%)	0.92	(0.90–0.94)	85%	≥30					0.84	(0.80–0.87)	78%	≥40	0.88	(0.85–0.91)	80%	≥33

The area under the curve (AUC) with 95% confidence interval (CI) describes a candidate score’s ability to classify patients who replied ‘completely recovered’ or ‘much improved’ on the GPE scale into ‘success’ and those replied ‘slightly improved’, ‘unchanged’, ‘slightly worse’, ‘much worse’, and ‘worse than ever’ into ‘non-success’ at 12 month follow-up. The larger the AUC, the better the accuracy of the score [range from 0.5 (no ability) to 1.0 (perfect ability)]. A cut-off corresponds to the threshold that gave rise to the maximum percentage of patients correctly classified (max corr. Class.) into ‘success’ and ‘non-success’. Results are given for ODI, EQ-5D, NRS leg pain, and NRS back pain for spinal stenosis and for degenerative spondylolisthesis. Because the EQ-5D questionnaire values ranged from −0.6 to 1.0 on a categorical scale, it was not mathematically possible to evaluate the percent change score

Except for the NRS back pain change score, the cut-off values for a clinically important outcome were identical for LSS and LDS (Table 3). The following cut-offs were estimated, with the correct classification rates (for LSS and LDS respectively) listed in parentheses:

### ODI

follow-up score ≤ 24 points (82%, 85%), change score ≥ 13 points (78%, 80%), percentage change ≥30% (83%, 85%).

### EQ-5D

follow-up score ≥ 0.692 points (78%, 84%), change score ≥ 0.105 points (73%, 76%). Because the EQ-5D questionnaire values ranged from − 0.6 to 1.0 on a categorical scale, it was not possible to find a mathematically adequate method to evaluate the percentage change score.

### NRS leg pain

follow-up score ≤ 3points (81%, 79%), change score ≥ 3 points (77%, 76%), percentage change ≥40% (81%, 78%).

### NRS back pain

follow-up score ≤ 4 points (82%, 83%), change score ≥ 2 points for LSS (75%) and ≥ 3 points for LDS (79%), percentage change ≥33% (80%, 82%).

The sensitivity and specificity for each cut-off value are listed in Table 4.

**Table 4:** Sensitivity and specificity for estimated cut-off values. Correct classification rate in different PROM baseline groups

	Spinal stenosis				Degenerative spondylolisthesis			
	Estimated cut-off	Correct classification	Sensitivity	Specificity	Estimated cut-off	Correct classification	Sensitivity	Specificity
ODI follow-up score	≤24		**0.83**	**0.80**	≤24		**0.85**	**0.84**
Low baseline		80%				87%		
Medium		85%				85%		
High baseline		80%				84%		
ODI change score	≥13		**0.78**	**0.77**	≥13		**0.83**	**0.71**
Low baseline		72%				77%		
Medium		84%				86%		
High baseline		78%				75%		
ODI percentage change	≥30		**0.87**	**0.77**	≥30		**0.89**	**0.77**
Low baseline		83%				88%		
Medium		85%				85%		
High baseline		80%				82%		
EQ-5D follow-up score	≥0.692		**0.76**	**0.81**	≥0.692		**0.80**	**0.88**
Low baseline		75%				81%		
Medium		79%				80%		
High baseline		80%				82%		
EQ-5D change score	≥0.105		**0.77**	**0.68**	≥0.105		**0.81**	**0.63**
Low baseline		73%				74%		
Medium		75%				80%		
High baseline		72%				71%		
Leg pain follow-up score	≤3		**0.80**	**0.83**	≤3		**0.79**	**0.78**
Low baseline		82%				81%		
Medium		82%				76%		
High baseline		81%				79%		
Leg pain change score	≥3		**0.78**	**0.75**	≥3		**0.78**	**0.72**
Low baseline		69%				70%		
Medium		82%				76%		
High baseline		78%				80%		
Leg pain percentage change	≥40		**0.82**	**0.80**	≥40		**0.80**	**0.73**
Low baseline		79%				75%		
Medium		81%				76%		
High baseline		81%				81%		
Back pain follow-up score	≤4		**0.84**	**0.79**	≤4		**0.85**	**0.79**
Low baseline		81%				82%		
Medium		83%				80%		
High baseline		82%				87%		
Back pain change score	≥2		**0.82**	**0.66**	≥3		**0.78**	**0.80**
Low baseline		72%				67%		
Medium		83%				81%		
High baseline		71%				83%		
Back pain percentage change	≥33%		**0.81**	**0.79**	≥33%		**0.83**	**0.78**
Low baseline		76%				78%		
Medium		83%				80%		
High baseline		80%				85%		

The sensitivity describes the probability of correctly classifying an individual replying ‘completely recovered’ or ‘much improved’ (GPE) as a ‘success’ when assessed by the estimated cut-offs for the PROMs. The specificity describes the probability for detecting a ‘non-success’ patient (one with a lower response at the GPE scale)

For each estimated cut-off values the percentage of correctly classified patients (correct classification) into ‘success’ and ‘non-success’ according to the anchor are given separately for patients with low (ODI; 0–32, EQ-5D; −0.59-0.1, NRS leg and back pain; 0–5), medium (ODI; 32–46, EQ-5D; 0.1–0.6, NRS leg and back pain; 6–7), and high (ODI; 46 to 100, EQ-5D; 0.6–1.0, NRS leg and back pain; 8–10) baseline scores

In the sensitivity analysis, a ≤ 24 point cut-off for the ODI follow-up score gave 80% correctly classified patients in low, 85% in medium and 80% in high baseline levels for LSS, respectively 87, 85 and 84% for LDS. The corresponding rates for the ODI change score were 72, 84 and 78% for LSS, and 77, 86 and 75% for LDS, and, for the percentage change score, 83, 85 and 80% for LSS, and 88, 85 and 82% for LDS. Table 4 shows that also for the other PROMs, the change scores for patients with low and high baseline values were the least accurate in matching ‘successes’ from the GPE scale.

## Discussion

We evaluated how accurately four frequently used PROMs would reflect the patients’ global assessment of being completely recovered or much better at 12-month follow-up. All outcome scores for the PROMs were highly correlated to the GPE score, indicating good predictive validity. The accuracy for correct classification of a GPE ‘success’ as a ‘success’ assessed by the PROMs was generally high, however, lower for the (numerical) change score than for the follow-up score and the percentage change score, especially among patients with low and high preoperative PROM values. All estimated cut-off values were the same for LSS and LDS, except for the change score for NRS back pain.

### Other studies

#### Follow-up score

In a study with a similar methodology to the present study, Fekete et al. [18] suggested that a follow-up score of ≤3 points is the best cut-off value for an acceptable level of leg pain and back pain following surgery for spinal stenosis with (*n* = 910) and without degenerative spondylolisthesis (*n* = 1625). This is in accordance with our estimate for leg pain and one point lower than our estimate for back pain. In a study [19] on patients with degenerative lumbar spine disorders operated with decompression (*n* = 1288), the estimated cut-off for ODI for a satisfactory symptom state was ≤22, nearly equivalent to our own criterion (≤24). Furthermore, they found the same cut-off estimates at 1-year and 2-year follow-up [19].

#### Change score

Carreon et al. [40] analysed patients operated with primary fusion surgery – 332 for spinal stenosis with spondylolisthesis (including both isthmic and degenerative cases) and 153 for spinal stenosis without spondylolisthesis. They evaluated the change score and found the minimum detectable change (smallest change above the upper limit of a 95% CI for the measurement error) to be 12.5 for ODI, 1.2 for NRS leg pain and 1.1 for NRS back pain. All these thresholds were below our estimated thresholds. Glassman et al. [17] found 18.8 for ODI, 2.5 for NRS leg pain and 2.5 for NRS back pain to be cut-offs for a substantial clinical improvement for patients (*n* = 357) treated with fusion surgery for several spinal disorders. Their ODI limit was higher than in our study, whereas their thresholds for pain were in accordance with our results.

The use of change scores for benchmarking has been criticized for not taking into account the patients’ baseline scores [12, 18, 41]. A numerical change from high baseline scores is probably of less importance than a change from low baseline scores.

In the present study, the change scores’ weak ability to correctly classify patients in the upper and lower baseline groups lends support to this criticism.

#### Percentage change score

In order to account for the influence of the baseline score on the outcome score, using the percentage change score has been recommended [12, 42]. Based on a literature review and an expert panel decision, Ostelo et al. [42] concluded that a > 30% change from baseline to follow-up was the best threshold for identifying clinically meaningful improvement in ODI and NRS back pain. Their cut-off for ODI is identical to our estimate, and their threshold for pain is in accordance with our estimate (> 33%). Dworkin et al. [12] suggested a 30% reduction in pain to be moderately important and a 50% reduction to be substantially important for patients treated for chronic pain. Our cut-off estimates for NRS leg- and back pain for LSS and LDS were between these suggestions.

### Methodical challenges

Because the EQ-5D questionnaire values ranged from − 0.59 to 1.0, it was not possible to adequately calculate the percentage change score. Hence, only the 12-month follow-up score and the change score could be provided for the EQ-5D.

### Application of the thresholds

As for other metrics developed for determining a clinically relevant outcome following treatment (i.e., MCID [8], (MIC) [27], a substantial clinical benefit [11] and a satisfactory symptom state [28]), it is essential to recognize that the thresholds from the present study cannot be directly applied to comparisons of mean outcome scores between groups [12, 13, 17, 43]. The thresholds are developed to determine whether an individual has achieved an important preoperative to postoperative benefit/improvement and should be used in the same context when comparing treatment effects [13]. Assuming a mean between-group difference in a PROM less than MCID to be clinically unimportant and a difference above MCID to be clinically important is warned against [12, 13, 43]. Instead the proportion of patients reaching the threshold for clinical improvement (the ‘success’ rate) should be calculated for each treatment group. Then the ‘success’ rates should be compared between the groups. This approach is advocated as a statistically and clinically useful tool for evaluating treatment effects [12, 16, 17, 24, 43, 44]. In discussion with patients, knowledge of the ‘success’ rate for a treatment can be employed as clinically relevant information in a shared decision-making process [17]. Furthermore, knowing the difference in the ‘success’ rates of two treatment groups makes it is possible to calculate the number needed to treat to obtain one extra patient with ‘success’ in an investigated group compared to a control group (NNT = 100 divided by the absolute difference in ‘success’ rate) [6, 12, 44]. For example, in patients with degenerative spondylolisthesis treated with either decompression alone or decompression with fusion, assessed by ODI, how many patients must be fused to get one more patient with a clinically relevant outcome? [6]. Finally, assumptions regarding the difference in the ‘success’ rate between groups provide the opportunity to estimate a statistically and clinically relevant sample size when planning a clinical trial [6, 12].

The proposed threshold from the present study is derived from populations with LSS and LDS. The threshold is condition-specific [13] and should be applied solely to these conditions.

### Strengths and limitations of the study

Strengths of this study are the large sample size and the collection of data through a comprehensive and well-structured registry. More than 90% of the national centres performing spinal stenosis surgery report to the registry, and currently more than 65% of operations for spinal stenosis are registered. The follow-up rate was good and in accordance with recommendations for spine registries [45].

For research on effectiveness and efficacy following treatment in a specific patient group it is recommended to use criteria for clinical improvement derived from populations similar to the one being studied [13]. The estimated thresholds derived from patients operated for LSS and LDS ensure reliable estimates for these conditions. Finally, we consider the evaluation of all scores in the same study and the consecutive sub-group analysis of the three baseline groups to be strengths.

There are several major limitations in the method used for determining the thresholds. As long as we know, the validity of a single-item rating (GPE scale) of how the patients are doing one year after spine surgery is not proven specifically for LSS and LDS. However there are some arguments in its favour. Using global assessment to evaluate patients’ satisfaction with treatment outcome in spinal disorders is recommended by international panels of experts in the field [12, 46, 47]. The global assessment of ‘pain free or much better’ and ‘much or very much improved’ has been considered to be an appropriate reference criterion for a successful outcome following spinal surgery [35]. In a Norwegian study of the validity of the GPE scale, the GPE replies were strongly associated with the follow-up scores for PROMs [48].

Another limitation is the evaluation of self-report measurements (ODI, EQ-5D, and NRS leg- and back pain) against another related self-report instrument (GPE) as a criterion [20]. Alternatively, an objective functional ‘non-self-report’ outcome, such as return to work, has been recommended [20]. However, this criterion is also criticized as return to work is not necessarily the primary goal for all patients, and it is not a relevant measurement for an elderly patient group [49]. Walking capacity is another criterion used to assess functional outcome in patients with spinal stenosis. In addition to asking about the walking distance before and after surgery, an objective assessment of walking distance could be recorded [50]. The differences in activity levels preoperatively and the patients expectations or anticipated activity level after surgery should also be taken into account. Patients’ who are happy to perform their limited activities of daily living, most probably accept more disability than patients involved in more demanding activities such as running and playing tennis. A suggested method, the ‘benefit-harm trade-off method’ [51, 52], in which the patients are asked to estimate how much benefit they would consider sufficient to justify the risk of getting worse after surgery, would take into account the patients’ accepted physical performance level. For the future this may be a suitable alternative approach for determining ‘success’- criteria.

The method used in the present study is described in detail and advocated by the ‘IMMPACT Recommendation’ [12], and is the most frequently used method for determining thresholds for clinical importance [17, 18, 25–28]. Furthermore, according to US FDA-recommended methodology for defining thresholds for PROMs, the GPE scale is considered a suitable anchor [29].

It is essential that the estimated PROM thresholds should be utilised and interpreted with caution. The evaluated PROMs do not assess all aspects that may be considered important for an individual. A patient who obtains an outcome in a PROM which exceeds the threshold for clinical importance may have non-observed complaints that are not detected; for example, loss of agility, slow walking speed and general stiffness of the back. Furthermore, objective data such as measured walking distance and muscle strength are not recorded in the registry questionnaire. Therefore, when reporting a ‘success’ rate it should be made clear that it is only an estimate of the proportion of patients reaching a threshold for improvement in a PROM considered to be of importance for a patient. An ideal PROM that covers all relevant domains of importance for all kind of patients will give a more accurate estimate of the ‘success’ rate.

## Conclusion

For estimating ‘success’ rates assessed by PROMs for patients operated for LSS and LDS we recommend using the follow-up score or the percentage change score. These scores reflect a clinically important outcome more accurately than the change score.

## Abbreviations

AUC: Area under curve
CI: Confidence interval
EQ-5D: EuroQol 5-dimensional questionnaire utility index
GPE: Global perceived effect
LDS: Lumbar degenerative spondylolisthesis
LSS: Lumbar spinal stenosis
NNT: Number needed to treat
NRS: Numeric rating scale
ODI: Oswestry disability index
PROMs: Patient reported outcome measurements
ROC: Receiver operating characteristics
